# *Helicobacter pylori* Eradication Therapy in Patients with Decreased Renal Function: A Systematic Review

**DOI:** 10.3390/jcm13030850

**Published:** 2024-02-01

**Authors:** Toshihiro Nishizawa, Masaya Sano, Osamu Toyoshima, Hidekazu Suzuki

**Affiliations:** 1 Department of Gastroenterology and Hepatology, International University of Health and Welfare, Narita Hospital, Narita 286-8520, Japan; nisizawa@kf7.so-net.ne.jp; 2 Gastroenterology, Toyoshima Endoscopy Clinic, Tokyo 157-0066, Japant@ichou.com (O.T.); 3 Division of Gastroenterology and Hepatology, Department of Internal Medicine, Tokai University School of Medicine, Isehara 259-1193, Japan

**Keywords:** *Helicobacter pylori*, eradication, decreased renal function, renal impairment, systematic review

## Abstract

**Background**: There are concerns that *Helicobacter pylori* eradication therapy may worsen kidney function in patients with decreased renal function. This study aimed to systematically review the literature regarding *Helicobacter pylori* eradication in patients with renal impairment. **Methods**: PubMed, the Cochrane Library, and Igaku Chuo Zasshi were searched for comparative studies on *H. pylori* eradication in patients with renal impairment. **Results**: Five articles were included in this systematic review. According to a randomized trial comparing a proton pump inhibitor (PPI) + clarithromycin + metronidazole and PPI + clarithromycin + amoxicillin in patients with decreased renal function, the incidence of acute renal failure was significantly lower in PPI + clarithromycin + metronidazole (2%: 1/44) than in PPI + clarithromycin + amoxicillin (18%: 8/44). The eradication rate in PPI + clarithromycin + metronidazole (92.5%) was significantly better than that in PPI + clarithromycin + amoxicillin (76.3%). According to four reports on eradication treatment using PPI + clarithromycin + amoxicillin in patients with and without decreased renal function, the eradication rates and adverse effects were similar in both groups. Regarding dose adjustment, three reports reduced the dose of antibiotics by half in patients with a creatinine clearance of 30 mL/min or less. **Conclusions**: The regimen with PPIs, clarithromycin, and metronidazole is recommended for renal impairment. The combination of PPIs, clarithromycin, and amoxicillin, at reduced doses depending on the renal function, is also a potential option.

## 1. Introduction

*Helicobacter pylori* (*H. pylori*) infection causes peptic ulcers and *H. pylori*-related diseases such as gastric cancer. *H. pylori* eradication is useful for the treatment and prevention of those diseases [1,2,3].

If patients with decreased renal function are infected with *H. pylori*, there are concerns regarding the impairment of renal function due to eradication treatments. Particularly, amoxicillin (AMPC) is a key drug for *H. pylori* eradication and can impair renal function [4].

Patients with decreased renal function should consider the selection of drugs and a reduction in drug dose.

Reports on eradication in patients with renal dysfunction are accumulating [5,6,7,8]. There is also a review of *H. pylori* eradication in patients undergoing dialysis [9]. Patients undergoing dialysis have exhausted their kidney functions; therefore, there is no longer any concern regarding the impairment of renal function. Rather, there is concern in patients with decreased renal function without dialysis. No systematic review of *H. pylori* eradication has been conducted in this context. And, appropriate eradication treatment for patients with decreased renal function is unclear. This study aimed to systematically search for studies on *H. pylori* eradication in patients with renal impairment and to suggest an appropriate eradication treatment. As a side note, this systematic review was conducted in response to one of the clinical questions in the 2024 revised edition of the Japanese Helicobacter Society Guidelines, “Which eradication treatment should be selected for patients with decreased renal function?”.

## 2. Method

### 2.1. Protocol

Before performing the meta-analysis, we developed a protocol in order to define the search strategies and determine the study selection criteria, as well as identify the methods for relevant data extraction and quality assessment. This systematic review was reported in accordance with the Preferred Reporting Items for Systematic Reviews and Meta-Analysis (PRISMA).

### 2.2. Eligibility Criteria

We systemically searched comparative studies on *H. pylori* eradication in patients with renal impairment. The inclusion criteria for this systematic review were as follows: (1) the study design was a comparative study; (2) the patients with decreased renal function received standard eradication treatment; (3) as a comparison group, the control group with normal renal function received standard eradication treatment or the patients with decreased renal function received other eradication treatments; (4) the eradication success rate or renal function before and after eradication was described; and (5) the age of the research participants was 16 years or older. The exclusion criteria were as follows: (1) research on patients undergoing dialysis, (2) case reports, (3) conference abstracts, (4) reviews, and (5) research protocols.

### 2.3. Literature Search Strategy

The literature was searched using PubMed, the Cochrane Library, and Igaku Chuo Zassh [10,11]. Two researchers (T.N. and H.S.) independently conducted the literature search and selection. If the results differed between the researchers, the differences were discussed. These results that were agreed upon by the two researchers were adopted.

### 2.4. Literature Search and Selection

The eligible papers were searched for using PubMed, the Cochrane Library, and Igaku Chuo Zasshi up to November 2023. The following words were used for the literature search: (*Helicobacter pylori* or *H. pylori*) and (eradication[Title/Abstract] or therapy[Title/Abstract]) and (kidney[Title/Abstract] or renal[Title/Abstract]) and (dysfunction or impairment or disease or failure or insufficiency or impaired function). We reviewed the titles and abstracts of the searched articles and excluded papers that did not meet the eligibility criteria. We then read the full texts of the remaining articles and included those that met the eligibility criteria for the systematic review.

### 2.5. Data Extraction

We prepared the standardized sheets for data abstraction. Extracted data included study design, research subject, *H. pylori* eradication treatments in the control group and the comparison group, outcomes, adverse effects, and study quality. The parameters of renal damage were serum creatinine and/or creatinine clearance (CCr) and the changes before and after eradication treatment.

### 2.6. Quality Assessment of Included Papers

Regarding non-randomized controlled trials, the quality of the research was evaluated based on the Newcastle–Ottawa Scale [12,13]. The Newcastle–Ottawa Scale consists of 4 items for selection, 2 items for comparability, and 3 items for outcome or exposure. The higher the total score, the higher the quality of the study. The highest score is 9. A score of ≥5 indicated research of good quality.

Randomized controlled trials (RCTs) were assessed based on The Cochrane Handbook for Systematic Reviews of Interventions [14,15]. Evaluation items included random sequence generation, allocation concealment, participant blinding, appropriate management of incomplete outcomes, avoidance of selective outcomes, and other concerns. If random sequence generation or allocation concealment was not described, the risk of bias was assessed as “unclear”. If blinding of participants and personnel was not performed, the risk of bias was assessed as “high”.

## 3. Results

### 3.1. Literature Selection

**Figure 1:**
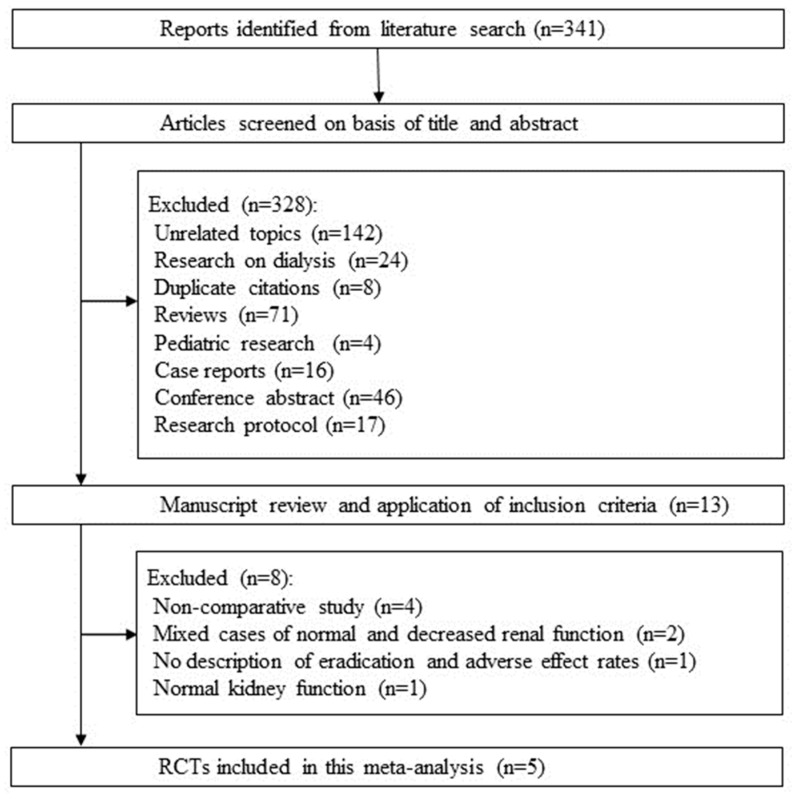
The flow of the literature included in the systematic review.

A total of 341 articles were identified, including 137 from PubMed, 95 from the Cochrane Library, and 109 from Igaku Chuo Zasshi. After screening the titles and abstracts, 13 papers remained. The remaining 13 papers were examined in detail. Four papers were excluded due to being a non-comparative study [16,17,18,19]. Two papers treated mixed cases of normal and decreased renal function and were excluded [20,21]. One study did not describe the rates of *H. pylori* eradication and adverse events and was excluded [22]. One study treated cases with normal CCr and was excluded [23]. After scrutiny, five papers were included in this systematic review [4,5,6,7,8] (Figure 1). There were two RCTs and three case-control studies.

### 3.2. Background and Methods in Included Studies

A summary of the included studies is presented in Table 1.

Seu et al. in Taiwan conducted an RCT for 88 patients with decreased renal function (serum creatinine > 1.5 mg/dL) and compared standard triple therapy with lansoprazole (LPZ), clarithromycin (CAM), and AMPC with triple therapy with LPZ, CAM, and metronidazole (MNZ) [4]. The treatment duration was 7 days, and the doses of LPZ, CAM, AMPC, and MNZ were 30 mg, 500 mg, 750 mg, and 500 mg twice a day, respectively. The doses were not adjusted based on the renal function. The means of serum creatinine were 2.7 ± 1.0 mg/dL in the LPZ, CAM, and MNZ group, and 2.7 ± 1.0 mg/dL in the LPZ, CAM, and AMPC group. At weeks 2 and 6 after the eradication, all patients received a serum creatinine test. Acute renal failure was defined as follows. The increase in serum creatinine was 0.5 mg/dL or greater for patients with a baseline creatinine level of 1.5–1.9 mg/dL; 1.0 mg/dL or greater for patients with a baseline creatinine level of 2.0–4.9 mg/dL; or 2 mg/dL or greater for patients with a baseline creatinine level of 5 mg/dL or higher. The total numbers of adverse events such as nausea, vomiting, diarrhea, and bitterness in the mouth were described in both groups. The success of the eradication was assessed via an *H. pylori*-specific stool antigen test 6 weeks after the eradication. The published year was 2003.

Seyyedmajidi in Iran conducted an RCT for 112 patients with different degrees of renal impairment and 50 control patients [5]. A 14-day standard triple therapy with omeprazole (OPZ), AMPC, and CAM was compared with a new sequential therapy with OPZ and AMPC both for 14 days, ciprofloxacin (CPFX) for the first 7 days, and furazolidone for the last 7 days. The doses of OPZ, AMPC, CAM, CPFX, and furazolidone were 20 mg, 1000 mg, 500 mg, 500 mg, and 200 mg twice a day, respectively. The doses of AMPC, CAM, and CPFX were reduced to 50% for patients with CCr < 30 mL/min. The numbers of cases with 30 mL/min ≤ CCr < 60 mL/min, CCr < 30 mL/min, and dialysis were 36, 37, and 39, respectively. Although patients with dialysis were included, the subgroup analyses by renal function were performed. It was also possible to compare cases with and without impaired renal function for standard triple therapy. Therefore, this systematic review included this study as a case-control study as well as a randomized controlled trial. The success of the eradication was assessed via a ^13^C-urea breath test 6 weeks after the eradication. The adverse events and the change in renal function were not described. The published year was 2011.

Alimadadi et al. in Iran reported a case-control study for 132 patients with *H. pylori* infection [6]. The eradication regimen was a 14-day standard triple therapy with OPZ, CAM, and AMPC. The doses of OPZ, CAM, and AMPC were 20 mg, 500 mg, and 1000 mg twice a day, respectively. The doses of CAM and AMPC were reduced to 50% for patients with CCr ≤ 30 mL/min. This study included patients with various levels of renal function from normal to dialysis. The numbers of cases with CCr ≥ 90 mg/dL, 60 mg/dL ≤ CCr < 90 mg/dL, 30 mg/dL ≤ CCr < 60 mg/dL, CCr < 30 mg/dL, without hemodialysis and with hemodialysis, were 26, 27, 26, 27, and 26, respectively. Although patients with dialysis were included, the subgroup analyses by renal function were performed. The success of the eradication was assessed via a ^13^C-urea breath test 6 weeks after the eradication. CCr was monitored for 3 months before the start of the study and during the study to ensure that it remained constant. However, the details about CCr monitoring were not described in the result section. The published year was 2015.

Liang et al. in Taiwan reported a case-control study for 758 patients with *H. pylori* infection [7]. The eradication regimen was a 7-day standard triple therapy with a proton pump inhibitor (PPI), CAM, and AMPC. The doses of PPI, CAM, and AMPC were 20 mg, 500 mg, and 1000 mg twice a day, respectively. The doses of CAM and AMPC were reduced to 50% for patients with end-stage renal failure. This study included patients with various levels of renal function from normal to dialysis. The numbers of cases with CCr ≥ 60 mg/dL (normal), 30 mg/dL ≤ CCr < 60 mg/dL, CCr < 30 mg/dL, without hemodialysis and with hemodialysis, were 628, 97, 17, and 16, respectively. Although patients with dialysis were included, the subgroup analyses by renal function were performed. The failure of the eradication was confirmed by either one positive result of the ^13^C-urea breath test or any two positive results of the rapid urease test, culture, and histology 4–8 weeks after the eradication. The adverse events and level of patient compliance were described. The definition of poor compliance was failure to finish 80% of all medication due to adverse events. The published year was 2017.

Mak et al. in China reported a case-control study for 42 patients with *H. pylori* infection [8]. The eradication regimen was a 7-day standard triple therapy with OPZ, CAM, and AMPC. The doses of OPZ, CAM, and AMPC were 20 mg, 500 mg, and 1000 mg twice a day, respectively. The doses were not adjusted based on the renal function. This study included patients with various levels of renal function from normal to dialysis. The numbers of cases with normal renal function, CCr < 30 mg/dL, without hemodialysis and with hemodialysis, were 21, 9, and 12, respectively. The baseline CCr was 21.4 ± 8.3 mL/min in patients with CCr < 30 mg/dL without hemodialysis. Although patients with dialysis were included, renal function before and after eradication was described in patients with decreased renal function without hemodialysis. Serum creatinine levels and CCr were measured 2 and 4 weeks after the eradication. CCr was calculated from 24 h urine creatinine excretion. The success of the eradication was confirmed by negative results of the histology from four biopsy specimens and a rapid urease test 4 weeks after the eradication. The published year was 2002.

### 3.3. Quality Evaluation

Regarding the quality of non-RCTs using the Newcastle–Ottawa Scale (nine-point scale), two papers received seven points, and one paper received six points (Table 1). All case-control studies selected hospital controls instead of community controls and did not adjust confounders. In one paper, renal function after eradication was measured only in the case group.

Quality assessments of the RCTs are shown in Table 2. None of the RCTs described random sequence generation or allocation concealment. However, all the papers were generally considered to have acceptable quality.

### 3.4. RCTs Comparing the Standard Treatment with Other Treatments in Patients with Decreased Renal Function

There were two RCTs on first-line standard treatments and other treatments for patients with decreased renal function (Table 3). Sheu et al. compared LPZ combined with CAM and AMPC with LPZ combined with CAM and MNZ in patients with serum creatinine levels of 2 to 5 mg/dL [4]. The incidence of acute renal failure was 18% (8/44) in the LPZ, CAM, and AMPC group and 2% (1/44) in the LPZ, CAM, and MNZ group. The incidence of acute renal failure in the LPZ, CAM, and AMPC group was significantly higher than in the LPZ, CAM, and MNZ group, and 7% (3/44) of the patients in the LPZ, CAM, and AMPC group required temporary dialysis. In two patients (4.5%), the serum creatinine levels did not return to the baseline. The adverse events in the LPZ, CAM, and AMPC group was more frequent than those in the LPZ, CAM, and MNZ group. The rate of complete drug compliance in the LPZ, CAM, and MNZ group (77.3%) was significantly higher than that in the LPZ, CAM, and AMPC group (52.3%; *p* < 0.05). The eradication rate in the LPZ, CAM, and MNZ group (92.5%) was also significantly higher than in the LPZ, CAM, and AMPC group (76.3%). They concluded that the combination of CAM and MNZ was more effective and safer.

Seyyedmajidi et al. compared a 14-day regimen of OPZ combined with CAM and AMPC with sequential therapy with OPZ combined with CAM, CPFX, and furazolidone as first-line eradication [5]. In patients with CCr < 60 mL/min, the eradication rates were 77.8% (28/36) for OPZ, CAM, and AMPC and 83.7% (31/37) for sequential therapy. No significant differences were observed between the groups. There was no description of adverse effects.

### 3.5. Standard Eradication Treatment for Patients with and without Decreased Renal Function

There were four reports of eradication treatment using PPIs, AMPC, and CAM in patients with and without decreased renal function [5,6,7,8] (Table 4). In every study, the eradication rates were similar in patients with and without decreased renal function. Liang et al. reported no significant differences in the adverse effects between patients with and without decreased renal function [7]. Mak et al. reported that the CCr before and after eradication did not change in the group with decreased renal function [8]. Liang et al. also reported that patient compliance was excellent in patients with and without decreased renal function (100% (130/130) and 99.8% (627/628)) [7].

For dose adjustment, one study used half the dose of CAM and AMPC when the CCr was below 30 mL/min [6]. Two studies used half the dose of CAM and AMPC in patients with end-stage renal failure [5,7]. One study did not reduce the dose of antibiotics but reported no change in CCr before and after eradication treatment [8].

## 4. Discussion

An RCT by Sheu et al. suggested that the combination of PPIs, CAM, and MNZ was more effective and safer than PPIs, CAM, and AMPC [4]. The potassium-competitive acid blocker vonoprazan (VPZ) has been reported to be superior to PPIs for *H. pylori* eradication [24,25,26,27]. In conclusion, the recommended regimen for patients with decreased renal function is VPZ, CAM, and MNZ. Both PPIs and VPZ are metabolized by the liver and do not require dose adjustment according to renal function. Another option is PPIs, AMPC, and CAM, with reduced doses depending on renal function. A half-dose of CAM and AMPC was safe when the CCr was <30 mL/min.

The combination of PPIs, CAM, and MNZ is an important option for patients allergic to penicillin. The Maastricht V/Florence consensus report also recommended the combination of PPIs, CAM, and MNZ for patients allergic to penicillin [28]. Several studies compared PPI combined with CAM and MNZ with VPZ combined with CAM and MNZ in patients allergic to penicillin. Sue et al. reported that the eradication rates were 100% (20/20) for VPZ combined with CAM, and MNZ and 83.3% (25/30) for PPI combined with CAM and MNZ [27]. Ono et al. and Adachi et al. also reported that VPZ improved the efficacy of *H. pylori* eradication therapy with a regimen consisting of CAM and MNZ [29,30]. However, there is little evidence for VPZ-based eradication in patients with decreased renal function.

MNZ and CAM are metabolized by the liver and AMPC is mainly excreted by the kidneys. For reference, we have cited general adjustments to the antibiotic doses based on renal function. According to the guidelines of the Japanese Society of Nephrology and Pharmacotherapy, adjustments to the antibiotic doses based on renal function are as follows [31]: the dose of CAM is 200 mg twice a day for CCr ≧ 10 mL/min, and 200 mg once a day for CCr < 10 mL/min; the dose of MNZ is 750–2250 mg/day for CCr ≧ 10 mL, and the half dose for CCr < 10 mL/min; the dose of AMPC is 250–500 mg three times a day for CCr ≧ 50 mL/min, 250–500 mg twice a day for CCr 10–50 mL/min, and 250–500 mg once a day for CCr < 10 mL/min; and the dose of CPFX is 600 mg/day for CCr ≧ 50 mL/min, 400 mg/day for CCr 10–50 mL/min, and 200 mg/day for CCr < 10 mL/min. According to the recommendations of the French Infectious Disease Society, AMPC and CAM should be adjusted for glomerular filtration rates (GFR) < 30 mL/min and MNZ should be adjusted for GFR < 15 mL/min [32].

The duration of the PPI, CAM, and MNZ treatment was 7 days, and the duration of PPI, CAM, and AMPC treatment was 7 or 14 days, according to the studies included in this systematic review. Although the Japanese guidelines for *H. pylori* eradication recommend 7 days as the treatment duration [1], the Maastricht VI/Florence consensus report recommends a treatment duration of 14 days for PPI-clarithromycin-based triple therapy [33]. The duration of PPI, CAM, and AMPC treatment should be determined based on the guidelines for each region.

A recent network meta-analysis reported the optimal first-line *H. pylori* treatments at global and regional levels [34]. Globally, 14-day levofloxacin (LVFX)-based sequential therapy was the most efficient. In regional levels, the most effective combinations were 10-day CAM-based sequential therapy for Africa, 14-day LVFX-based sequential therapy for Asia, and 14-day CAM-based triple therapy for Europe. On the other hand, the Maastricht VI/Florence consensus report recommends bismuth quadruple therapy (PPI, bismuth, tetracycline, and MNZ) or non-bismuth concomitant quadruple therapy (PPI, CAM, AMPC, and MNZ) as a first-line eradication treatment in the areas of high (>15%) or unknown CAM resistance. In the areas of low CAM resistance, bismuth quadruple therapy or triple therapy with PPI, CAM, and AMPC is recommended as first-line empirical treatment. Bismuth is reported to have nephrotoxicity in patients with renal impairment [35], and it might be safer to avoid bismuth. The generally recommended first-line eradication regimen should be modified in patients with decreased renal function.

Eradication therapy occasionally causes irreversible adverse effects on renal function; therefore, it is necessary to consider the balance between risks and benefits when administering eradication therapy. One strategy to avoid these risks is to withhold *H. pylori* eradication until dialysis is initiated. However, several reports have indicated that *H. pylori* eradication may have protective effects on renal function [18,36]. However, this is not yet definitive and requires further investigation.

This systematic review had several limitations. The literature was limited, with only two RCTs and three case-control studies. Therefore, it is difficult to draw conclusive treatment conclusions for renal impairment. Further, large-scale studies are warranted to validate the efficacy of PPIs, CAM, and MNZ in patients with renal impairment. The included studies did not contain data on drug susceptibility testing. The regimen of PPIs, CAM, and MNZ may not be fully effective, depending on the region or race. A drug susceptibility-guided strategy for the treatment of renal impairment is a future issue.

## 5. Conclusions

A regimen of PPIs, CAM, and MNZ has been suggested for the treatment of renal impairment. PPIs, CAM, and AMPC at reduced doses, depending on the renal function, are also options. However, indications for eradication treatment should be carefully determined.

## Figures and Tables

**Table 1:** Characteristics of studies included in the systematic review.

First Author	Study Design	Subjects	Comparison Group	Control Group	Outcomes	NOS
Sheu [4]	Randomized controlled trial	*H. pylori*-infected cases with renal dysfunction	PPI + MNZ + CAM	PPI + AMPC + CAM	Eradication rate, renal impairment	-
Seyyedmajidi [5]	Randomized controlled trial	*H. pylori*-infected cases with renal dysfunction	PPI + AMPC + CPFX + furazolidone	PPI + AMPC + CAM	Eradication rate	-
Alimadadi [6]	Case-control study	*H. pylori*-infected cases	PPI + AMPC + CAM in cases with decreased renal function	PPI + AMPC + CAM in cases with normal renal function	Eradication rate	7
Liang [7]	Case-control study	*H. pylori*-infected cases	PPI + AMPC + CAM in cases with decreased renal function	PPI + AMPC + CAM in cases with normal renal function	Eradication rate, adverse event	7
Mak [8]	Case-control study	*H. pylori*-infected cases	PPI + AMPC + CAM in cases with decreased renal function	PPI + AMPC + CAM in cases with normal renal function	Eradication rate, renal impairment	6

NOS: Newcastle–Ottawa Scale.

**Table 2:** Bias evaluation of studies included in the systematic review.

First Author	Random Sequence Generation	Allocation Concealment	Blinding of Participants and Personnel	Blinding of Outcome Assessment	Adequate Assessment of Incomplete Outcome	Selective Reporting Avoided	No Other Bias
Sheu [4]	Unclear	Unclear	High risk	Low risk	Low risk	Low risk	Low risk
Seyyedmajidi [5]	Unclear	Unclear	High risk	Low risk	Low risk	Low risk	Low risk

Unclear: there was no description of random sequence generation or allocation concealment.

**Table 3:** Randomized trial comparing standard triple therapy and new therapy in cases with decreased renal function.

First Author	Subjects	Treatments	Duration	Eradication Rate (PP)	Renal Impairment
Sheu [4]	Serum Cr > 1.5 mg/dL Non-dialysis	LPZ(30 mg) + MNZ(500 mg) + CAM(500 mg) bid	7 days	92.5% (37/40)	2% (1/44)
		LPZ(30 mg) + AMPC(750 mg) + CAM(500 mg) bid	7 days	76.3% (29/38) *	18% (8/44) *
Seyyedmajidi [5]	CCr < 60 mg/min Non-dialysis	Sequential therapy: OPZ (20 mg) + AMPC (1000 mg) + CPFX (500 mg: first half) + furazolidone (200 mg: second half) bid	14 days	83.7% (31/37)	-
		OPZ(20 mg) + AMPC(1000 mg) + CAM(500 mg) bid	14 days	77.8% (28/36)	-

PP: Per protocol, Cr: creatinine, CCr: creatinine clearance, *: *p* < 0.05, LPZ: lansoprazole, OPZ: omeprazole, CPFX: ciprofloxacin, bid: twice a day.

**Table 4:** Standard triple therapy (PPI + AMPC + CAM) in cases with decreased renal function.

First Author	Duration	Treatment Groups	Antibiotic Dose Adjustment	Eradication Rate (PP)	Adverse Events
Alimadadi [6]	14 days	Decreased renal function	Half dose for CCr ≦ 30 mL/min	81.2% (43/53)	-
		Normal renal function	None	83.0% (44/53)	-
Liang [7]	7 days	Decreased renal function	Half dose for end-stage renal disease	85.1% (97/114)	3.1% (4/130) ^#^
		Normal renal function	None	85.7% (538/628)	4.6% (29/628)
Mak [8]	7 days	Decreased renal function	None	90.5% (19/21) ^##^	CCr did not change before and after eradication
		Normal renal function	None	85.7% (18/21)	-
Seyyedmajidi [5]	14 days	Decreased renal function	Half dose for end-stage renal disease	77.8% (28/36)	-
		Normal renal function	None	76.0% (19/25)	-

CCr: creatinine clearance, ^#^: The analysis of adverse events includes 16 cases of dialysis, ^##^: The analysis of eradication rates includes 12 cases of dialysis.

## Data Availability

No additional data are available.
